# Untreated Stroke as Collateral Damage of COVID-19: “Time Is Brain” Versus “Stay at Home”

**DOI:** 10.1177/1941874420929199

**Published:** 2020-05-26

**Authors:** David Z. Rose, W. Scott Burgin, Swetha Renati

**Affiliations:** 1 Department of Neurology, Morsani College of Medicine, 7831 University of South Florida, Tampa, FL, USA

**Keywords:** stroke, acute stroke therapy, dysgeusia, anosmia, COVID-19

## Abstract

For decades, neurologists have been advocating that anyone with acute focal deficits report immediately to the closest hospital’s emergency room. Major advancements in the hyperacute diagnosis and treatment of stroke have justified our call-to-action slogan of “Time is Brain”—faster therapy leads to superior outcomes. However, this mantra has been recently usurped by the catchphrase “Stay at Home” during the coronavirus disease 2019 (COVID-19) pandemic. Fewer patients are presenting to hospitals with acute stroke; our census is down. Presumably the etiology of this phenomenon is either strict “social distancing” that some people may misperceive to exclude even emergent situations, or fears of contracting the virus while hospitalized. In this Short Report, we describe the year-over-year drop in stroke volume (ischemic and hemorrhagic both) coinciding with a paradoxical rise in acute reperfusion therapies at our university hospital. These data imply that stroke patients with mild/moderate symptoms are most likely staying home, and not receiving urgent therapies, and correspondingly, only the most severely disabled stroke patients are ultimately seeking and receiving help. We must remind our patients and the general public that our services are essential and available, as stroke still remains a medical emergency, and carries a likely higher overall mortality risk than COVID-19. As neurologists, we also must be vigilant for the atypical presentations and varied etiologies of stroke associated with COVID-19 as well.

## Short Report

An unforeseen consequence of the COVID-19 pandemic is the sudden decrease in patients presenting to the emergency room with acute stroke. Despite our panoply of advancements in the hyperacute diagnosis and treatment of stroke, several comprehensive cerebrovascular centers have recently observed an abrupt drop in the census of their stroke service. The etiology of this presumably is either strict interpretation of “social distancing” that some people may misperceive to exclude even emergent situations, or fears by patients or caregivers of contracting the virus while hospitalized. Our longstanding call-to-action mantra of “Time is Brain” has been usurped by “Stay at Home.”

In Florida, 20.5% of the population is 65 years of age or older, and many states have a similarly sizeable elderly demographic. This group represents the highest COVID-19 risk and simultaneously the highest stroke risk. Noticing an obvious decline in our stroke census at our university-based hospital in Tampa, we tabulated our numbers this year since the virus began spreading rapidly in February and into March, and compared this data to the mean number of stroke patients we saw during February and March of 2017, 2018, and 2019. The mean number of patients during those 2-month epochs in those years was 230; however so far in 2020, we saw only 194 during these 2 months, and April has trended even lower. Paradoxically, however, the rate of stroke alerts at our facility during this period that received acute reperfusion therapy—either intravenous tissue plasminogen activator or endovascular clot retrieval has risen to 29%—significantly higher than the average rate of 21% seen between 2017 and 2019. This implies that stroke patients with mild or moderate symptoms are most likely “Staying at Home” as only the most severely disabled stroke patients are ultimately seeking help—and receiving it.

As our understanding of this pandemic evolves, data from China, Italy, and other countries suggest that we must remain vigilant for atypical presentations of stroke, and also for its varied etiologies: it is hypothesized that COVID-19 may disrupt coagulation,^1^ provoke more large-vessel occlusions and cardioemboli, and even invade the central nervous system directly via the olfactory neuroepithelium, propagating from within the olfactory bulb to provoke symptoms of anosmia and dysgeusia.^2^

To adapt to this pandemic, stroke centers nationwide are adjusting acute stroke response algorithms, order sets, and protocols,^3^ expanding telehealth (both inpatient and outpatient) and shifting most nonemergent inpatient testing assessments (such as transesophageal echocardiography and extended cardiac monitoring, for example) into the outpatient arena. Such temporary modifications may have trifold benefit: limiting aerosolized procedures, decreasing length of stay, and opening up beds for COVID-19 patients—of major benefit when infection rates peak in each locality.

It is incumbent upon us as neurologists to remind the public that stroke is a bona fide medical emergency and that the risk of deferring such care more likely could be lethal than COVID-19: the mortality risk for ischemic stroke is about 15% and for hemorrhagic is about 42%—both significantly exceed that for COVID-19, currently estimated at approximately 1% to 3%.^4,5^ Stroke should not be disregarded or marginalized during a pandemic. The public needs reassurance now more than ever that hospitals have units for COVID-19 patients already preprepared and separate from everyone else—so that emergencies such as stroke (and heart attack) can still receive optimal treatment in a timely and safe manner. This may mitigate the COVID-19 collateral damage of at-home stroke that forfeits top-line treatment during the pandemic. By modifying best practices in the hospital and amplifying this message to the public, we can encourage highest risk individuals to realize that our vital slogan of “Time is Brain” supersedes “Stay at Home.” Stroke treatment is an essential service and we remain open for business.
